# Large Phenotypic Variation of Individuals from a Family with a Novel *ASPM* Mutation Associated with Microcephaly, Epilepsy, and Behavioral and Cognitive Deficits

**DOI:** 10.3390/genes13030429

**Published:** 2022-02-25

**Authors:** Randi von Wrede, Martin Schidlowski, Hans-Jürgen Huppertz, Theodor Rüber, Anja Ivo, Tobias Baumgartner, Kerstin Hallmann, Gábor Zsurka, Christoph Helmstaedter, Rainer Surges, Wolfram S. Kunz

**Affiliations:** 1 Department of Epileptology, University Hospital Bonn, 53127 Bonn, Germany; martin.schidlowski@ukbonn.de (M.S.); theodor.rueber@ukbonn.de (T.R.); anja.ivo@ukbonn.de (A.I.); tobias.baumgartner@ukbonn.de (T.B.); gabor.zsurka@ukbonn.de (G.Z.); christoph.helmstaedter@ukbonn.de (C.H.); rainer.surges@ukbonn.de (R.S.); 2 German Center for Neurodegenerative Diseases (DZNE), 53127 Bonn, Germany; 3 Swiss Epilepsy Clinic, Klinik Lengg AG, 8008 Zurich, Switzerland; hans-juergen.huppertz@kliniklengg.ch; 4 Department of Experimental Epileptology and Cognition Research, University Hospital Bonn, 53127 Bonn, Germany; kerstin.hallmann@ukbonn.de

**Keywords:** primary hereditary microcephaly, *ASPM* mutation, epilepsy, behavioral and cognitive deficits

## Abstract

Here, we report a consanguineous family harboring a novel homozygous frame-shift mutation in *ASPM* leading to a truncation of the ASPM protein after amino acid position 1830. The phenotype of the patients was associated with microcephaly, epilepsy, and behavioral and cognitive deficits. Despite the obvious genetic similarity, the affected patients show a considerable phenotypic heterogeneity regarding the degree of mental retardation, presence of epilepsy and MRI findings. Interestingly, the degree of mental retardation and the presence of epilepsy correlates well with the severity of abnormalities detected in brain MRI. On the other hand, we detected no evidence for substantial nonsense-mediated *ASPM* transcript decay in blood samples. This indicates that other factors than ASPM expression levels are relevant for the variability of structural changes in brain morphology seen in patients with primary hereditary microcephaly caused by *ASPM* mutations.

## 1. Introduction

Mentally disabled patients suffering from epilepsy, and especially from drug-resistant epilepsy, need an exact and widespread work up, including genetic testing, as clear diagnosis helps the patients and their caregivers to understand and accept the diagnosis. Furthermore, it helps the treating clinician to find appropriate treatment regimens as well as prevents initiation of useless treatments [1,2].

Microcephaly, primary hereditary (MCPH), is a rare neurodevelopmental disorder characterized by microcephaly and intellectual disability [3] in absence of other congenital abnormalities [3,4]. *ASPM* (abnormal spindle-like microcephaly-associated) gene mutations (almost exclusively nonsense, frame-shift or splice site mutations) are the most frequent underlying genetic cause for autosomal recessive MCPH [3]. The *ASPM* gene maps at the locus 1q31.3.; it consists of 28 exons and encodes a protein that consists of 3477 amino acids. Animal data confirm the important role of ASPM in cell cycle division of neural progenitor cells and support the idea of ASPM dysfunction leading to a neuronal developmental dysfunction (for an overview see [5]). Seizures are found in up to 15% of ASPM-MCPH patients [6,7] and should lead to a diligent MRI work up.

Here, we report a consanguineous family with three siblings harboring a novel homozygous *ASPM* frame-shift mutation causing protein truncation after amino acid position 1830. Despite the obvious genetic similarity, all three patients show a considerable phenotypic heterogeneity regarding the degree of mental retardation, presence of epilepsy and MRI findings. These differences are unlikely related to differences in *ASPM* expression, since increased mutant *ASPM* expression in blood was detected in the most affected patient in comparison to controls and heterozygous carriers.

## 2. Materials and Methods

### 2.1. Subjects

Clinical information was derived from actual datasets collected at in- and outpatient appointments at the University Hospital Bonn, Department of Epileptology, in 2019 and 2021, as well as clinical information from previous examination. Written informed consent for publication of clinical data and clinical information was given by the caregiver.

### 2.2. Neuropsychological Rating

Two tests for estimation of intelligence were performed: Raven’s Progressive Matrices (RPM) [8], which is a nonverbal assessment of “general cognitive ability” in terms of inductive and deductive reasoning or ”meaning making”, reported as an Intelligence Quotient, and the Kaufman Assessment Battery for Children (Second Edition) [9], which covers a comprehensive range of skills, including sequential and simultaneous processing, learning, problem solving and crystalline skills, which are important for understanding children with learning difficulties or psychological problems.

Three scales were used for assessing severity of disability: the Global Assessment of Severity of Epilepsy Scale (GASE) [10], the SINGER scale [11] and the modified Rankin scale (mRS) [12]. mRS is a single-item, seven-point ordinal scale for clinicians ranging from zero (no disability) to six (death). Global Assessment of Severity of Epilepsy Scale (GASE) is a single-item, seven-point global rating scale to assess disease severity ranging from one (not severe) to seven (extremely severe) considering all aspects of a patient’s life with epilepsy. The SINGER scale assesses 20 functions including self-care, mobility and cognition, which are rated from zero (totally dependent on professional help) to five (independent without any assistance). A physical activity score, a cognition score, a household score and a total score can be extracted, as well as the Barthel Index. Individual SINGER outcomes are reported as percent achieved in regard to 100% independency. For SINGER physical activity, >75% is rated as “mild/no impairment”, 47–75% as “moderate impairment” and below 46% as “severe impairment”. For SINGER cognition, >86% is rated as “no/mild impairment”, 31–86% as “moderate impairment” and below 31% as “severe impairment”. For SINGER household, >80% is rated as “no/mild impairment”, 40–60% as “moderate impairment” and below as 40% as “severe impairment”.

### 2.3. Whole Exome Sequencing

Genomic DNA was isolated from blood by routine techniques. The index patient’s DNA was enriched for exons using the Agilent SureSelect Human All Exon V6 kit (Agilent Technologies, Santa Clara, CA, USA). Paired-end reads of 100 bp resulted in a mean coverage of 82-fold (30-fold coverage for 78.5% and 10-fold coverage for 94.3% of target sequences). Reads were mapped and variants annotated as described [13]. Filtering and variant prioritization was performed using the VARBANK database and analysis tool at the Cologne Center for Genomics. In particular, we filtered for high-quality (coverage > 15-fold; phred-scaled quality > 25), rare homozygous variants (MAF ≤ 0.01 based on gnomAD; [14]) with predicted effects on protein sequence or splicing. To exclude pipeline-related artifacts, we filtered (MAF ≤ 0.01) against variants from in-house WES datasets from 511 epilepsy patients. Direct sequencing of purified PCR products was performed by a commercial service (Eurofins, Ebersberg, Germany).

### 2.4. ASPM Expression Analysis

Total blood RNA was obtained with the PAXgene system (Qiagen, Hilden, Germany). Complementary DNA (cDNA) was produced from RNA templates with the iScript Select cDNA synthesis kit (Bio-Rad Laboratories, Munich, Germany). A 1646 bp cDNA fragment between *ASPM* exon 17 and exon 18 was amplified using primers 5′-CAT CAC TTA TTC AGG GAT ATT G-3′ and 5′- TTG ATG TTC CCT TCT AAT CTG T -3′. Amplification was performed using RANGER DNA polymerase, under the following condition: 5 min at 95 °C; 35 cycles of 30 s at 95 °C, 25 s at 55 °C and 3 min at 68 °C; and finally, 10 min at 68 °C.

### 2.5. MRI Processing and Analysis

T1-weighted 3D whole-brain MR images of the three subjects (subjects II.2/II.4/II.3 with 1 mm/0.5 mm/0.8 mm isotropic resolution) were segmented into white matter, gray matter and CSF. Volumes of brain structures, including cerebrum and cerebellum, were determined by atlas-based volumetry (ABV), a fully automated, observer-independent method for volumetric analysis of MR images using algorithms of SPM12 and masks of diverse brain atlases [15,16]. The resulting volumes were adjusted by intracranial volume (ICV). ICV-normalized results were compared with an age-matched control group to obtain Z-scores. Additionally, a cortical reconstruction was performed, facilitating a gray matter surface and thickness assessment, using the recon-all pipeline of the Freesurfer [17] image analysis suite (version 7.1.1). Intermediate and final registrations and segmentations of Freesurfer analysis were inspected and manually corrected if required.

## 3. Results

**Figure 1:**
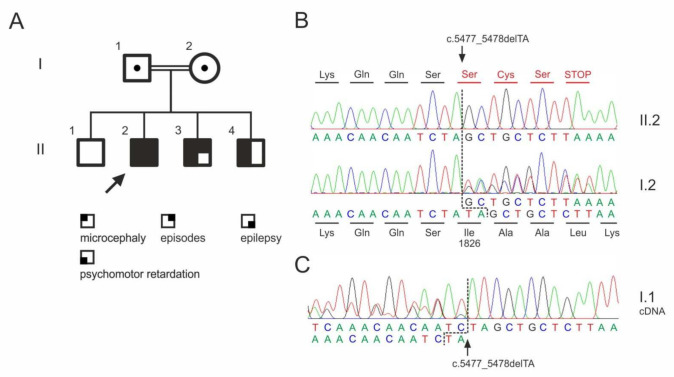
*ASPM* mutation in a consanguineous family. (**A**) Pedigree. Dots indicate heterozygous carriers. No material was available from the unaffected sibling (II.1) for genotyping. (**B**) Sequencing chromatograms of the index patient (II.2) and his unaffected mother (I.2). (**C**) Sequencing chromatogram from cDNA obtained from whole blood RNA sample, confirming the heterozygous mutation in the father (I.1). Please note the presence of the mutant transcript in the sequencing chromatogram showing the absence of significant nonsense-mediated decay.

We report on three affected sons of a consanguine relation (the parents are first degree cousins), Figure 1A. A fourth, 23-year-old son (II.1) was not affected. The parents are clinically unaffected, respectively. The family originally comes from Iraq. Samples from the unaffected brother (II.1) were not available.

### 3.1. Case A (II.2)

This 21-year-old man was referred to us at the age of 19 years because of drug-resistant epilepsy. He was born after an uneventful pregnancy to healthy parents.

Already in early childhood, moderate psychomotor delay became obvious with left-side mild spastic hemiparesis, leading to inability to walk as late as at the age of four. A global delay of childhood development was reported by the parents, with first words at the age of five. Apart from shyness, no behavioral abnormality impairing everyday life was reported by the parents. Difficulties in falling asleep and a shift in the day-night rhythm were reported with a moderate impact on everyday life. At the age of ten, he started to display focal impaired awareness seizures as well as focal impaired awareness seizures to bilateral tonic-clonic seizures. Initially treated with carbamazepine monotherapy, add-on with levetiracetam did not achieve long-term seizure freedom. At the first appointment, we saw a moderately mentally disabled young man with microcephalus, with mild left spastic hemiparesis of normal height. No other congenital abnormalities were found.

He was suffering from weekly seizures. Further antiseizure treatment regimen including valproate and oxcarbazepine were unsuccessful. A 24 h long-term EEG recording displayed right central and temporal epileptiform discharges and three similar focal impaired awareness seizures showing an ictal pattern frontocentral, once accentuated in the right hemisphere. This high seizure frequency within 24 h with adequate blood levels of antiseizure medication as well as the registered mild semiology of seizures suggests a higher seizure frequency as reported by the family.

### 3.2. Case B (II.3)

This 19-year-old man was referred to us at the age of 17 years as epilepsy was suspected. He was born at the expected date after an uneventful pregnancy to healthy parents. In early childhood, mild psychomotor delay was reported by the parents. Moderate behavioral abnormality including aggression and reduced tolerance to frustration leading to intrafamiliar distress was the main problem reported by the parents. Difficulties in falling asleep were reported, though without impact on everyday life. According to his parents, at the age of eighteen, he started to display weekly episodes with non-responsiveness and gazing for about 30 seconds; initial treatment with levetiracetam monotherapy did not lead to freedom of episodes. Episodes have never been observed at school or elsewhere.

At the first appointment, we saw a slightly mentally disabled young man with microcephalus and reduced body height (third percentile); no other congenital abnormalities were found. He was suffering from weekly episodes. A 96 h long-term EEG recording displayed neither epileptiform discharges no epileptic seizures. Epilepsy could not be verified.

### 3.3. Case C (II.4)

This 16-year-old boy who was referred to us at the age of 16 years to test for epilepsy. He was born at expected date after an uneventful pregnancy to healthy parents. In early childhood, mild psychomotor delay was reported by the parents. He has never displayed episodes which were suspicious for seizures. Behavioral abnormality including irritability and easy frightening was reported, although with minor impact on everyday life. Moreover, difficulty sleeping through the night was reported, although this only had a mild impact on everyday life.

At the first appointment, we saw a mild mentally disabled boy with microcephalus and of normal height. No other congenital abnormalities were found. He was not suffering from any episodes. A 96 h long-term EEG recording displayed neither epileptiform discharges no epileptic seizures. Epilepsy could not be verified.

### 3.4. Cognitive and Disability Assessment of the Individual Patients

The cognitive function and the degree of disability of the individual patients in the family (II.2, II.3, II.4; Figure 1A) were assessed by tests of intelligence (intelligence quotient) and standardized behavioral rating scales (Table 1). While the global intellectual level in all three patients turned out to be very low (<intelligence quotient 55), the patients in part differed considerably in regard to their independency on others as defined according to the international classification of functioning related ratings. Accordingly, patient II.2 showed epilepsy and the highest overall impairment, while patient II.3 was affected to a much lesser degree.

### 3.5. Whole Exome Sequencing

Whole exome sequencing in the index patients revealed a previously not described homozygous mutation in the *ASPM* gene (NM_18136.5:c.5477_5478del; p.Ile1826Serfs*4), which was verified by Sanger sequencing (Figure 1B). The mutation classifies as pathogenic according to the criteria by the American Collage of Medical Genetics (PVS1, PM2, PM3; [18]). The two affected brothers were also homozygous for the mutation. The clinically unaffected parents were heterozygous carriers.

**Figure 2:**
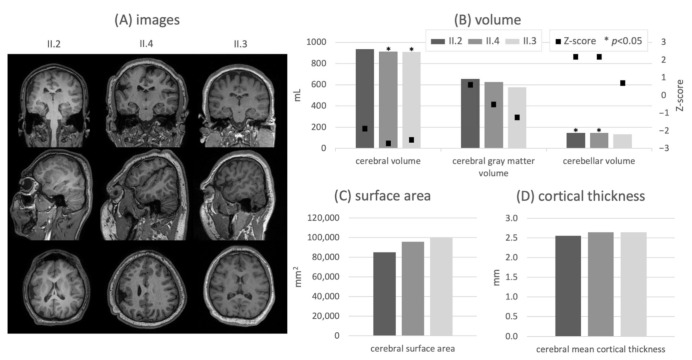
T1-weighted MR images in all orientations with a sagittal view of the right hemisphere (**A**), ICV-normalized volumes and corresponding Z-scores obtained by comparison with an age-matched control group per subject (**B**), brain surface area (**C**) and cortical gray matter thickness (**D**) for all subjects. The quantitative results are consistent with the MR abnormalities and match the highest disease severity seen in patient II.2. * *p* < 0.05.

As a result of the deletion of two bases, the reading frame in the *ASPM* gene shifts from codon 1826 and leads to a premature termination of the protein synthesis (Figure 2B). The other homozygous variants detected in the index patient (Table 2) were either described in ClinVar as benign variants (*APP*) or were present in homozygous state in controls.

Interestingly, the *ASPM* frame shift mutation does not cause nonsense-mediated transcript decay as visible in the cDNA sequencing chromatogram from the heterozygous father (I.1) (Figure 2C).

### 3.6. MRI Processing and Analysis

Radiological assessment showed microcephaly and microgyria in the right hemi-spheres, which were most pronounced in subjects II.2 and II.4. These abnormalities are evident in the quantified measures: All three subjects show a reduced ICV compared to the age-matched control group (Z-scores of subjects II.2/II.4/II.3: −5.1/−3.9/−4.0, *p* < 0.01). Thereby, the subject with the highest severity of cognitive impairment presents the strongest deviation. Likewise, ICV-normalized results (Figure 2B) indicate a subaverage expression of the cerebrum (Z-scores of subjects II.2/II.4/II.3: −1.9/−2.7/−2.5). In contrast, a volumetric overrepresentation of the cerebellum relative to the control group is apparent (positive Z-scores for subjects II.2/II.4/II.3: +2.2/+2.2/+0.7). No significant difference in cerebral relative gray matter volume relative to the control group is found. The volumetric results are complemented by corresponding brain surface area characteristics (Figure 2C), which show a clearly reduced cortical surface according to the degree of cognitive disability. Again, the strongest reduction is visible in the most impaired subject. The same relationship appears in the cortical thickness distribution (Figure 2D), as a decrease of the cerebral mean cortical thickness is present in the most severely affected subject (II.2).

## 4. Discussion

**Figure 3:**
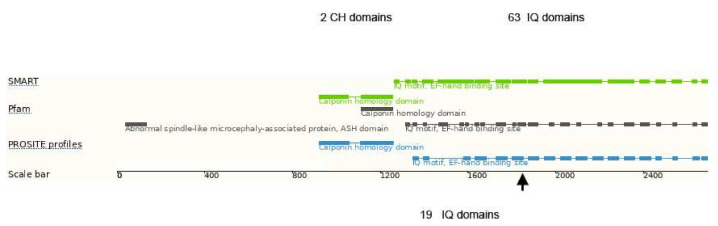
Domain structure of the human ASPM protein. CH domains–calponin homology domains, IQ domains–isoleucine-glutamine domains. The arrow indicates the truncation position due to the homozygous *p*.Ile1826Serfs*4 mutation, leaving 19 intact IQ domains. Modified according to [21].

ASPM is a microtubule-associated protein found at the spindle poles and centrosomes and is essential for normal mitotic spindle function in embryonic neuroblasts of developing CNS. In humans, it comprises 63 IQ domains (the single letter code for the amino acids isoleucine and glutamine), which mediate interaction with the EF-hand motifs of calmodulin- and calmodulin-related proteins (Figure 3) [20] and are believed to be essential for cerebral cortical neurogenesis.

In earlier work, *ASPM* expression, in particular the number of IQ domains, were suggested to be correlated with cortical development and it is discussed as one of the essential genes important for the increase of the cortical size during primate evolution [22,23,24]. Mutations in *ASPM*, the most common recessive microcephaly gene, reduce the cortical volume by at least 50% in humans, but have little effect on the brains of mice, which probably reflects evolutionarily divergent functions of this protein. The major role of ASPM in the early cortical development and gyration has been later confirmed in *Aspm* knockout ferrets [20], which are a better model than mice for the cortical gyration pattern of the human brain. The authors propose an evolutionary mechanism by which ASPM regulates cortical expansion during evolution by controlling the affinity of ventricular radial glial cells for the ventricular surface, thus modulating the ratio of ventricular radial glial cells, the most undifferentiated cell type, to outer radial glia, a more differentiated progenitor. Accordingly, differences in the phenotypic expression of available *ASPM* transcripts leading to different cortical surface areas have been attributed to the large phenotypic variability of primary hereditary microcephaly (MCPH) patients due to *ASPM* mutations. This transcript variability could be caused by a different degree of nonsense-mediated decay of *ASPM* transcripts harboring nonsense mutations. This would explain why so far, only nonsense, splice-site or frame-shift mutations in this particular gene have been associated with autosomal recessive MCPH caused by *ASPM* mutations [25]. In contrast to this, we have observed in blood samples no evidence for a substantial nonsense-mediated *ASPM* transcript decay. Therefore, the missing non-functional protein with the appropriate amount of IQ domains (Figure 3) seems to be essential to explain the fact that even a small deletion in the C-terminus is sufficient to cause severe microcephaly [5]. Thus, a decrease of IQ domain numbers in the potentially expressed truncated forms of the protein, rather than a decrease of its total amount, might be relevant for the dramatic decrease of cerebral surface area and the reduced cortical gyration pattern of MCPH patients.

The three brothers with microcephaly and mental retardation reported here carry a novel homozygous *ASPM* mutation, leading to a premature termination of the ASPM protein at amino acid position 1830. As indicated in Figure 3, this truncated ASPM protein contains only 19 IQ domains. All affected brothers show a significantly reduced cerebral volume compared to sex- and age-matched controls. Though carrying the same loss-of-function mutation at a very similar genetic background (male siblings from consanguineous parents), the phenotype differs substantially between the affected patients: one individual (II.2) exhibited severe epilepsy, high degree of mental retardation and a low cortical surface area, while his sibling (II.3) had no epilepsy, only mild psychomotor delay and an almost normal cerebral surface area. A rather high intrafamiliar phenotype variability in *ASPM* mutation in MCPH patients has been mentioned before [5], but no reasons were identified.

These large phenotypic differences are unlikely to be related to differences in *ASPM* transcript levels due to different degrees of nonsense-mediated decay of the mutated *ASPM* transcript. This result does not exclude potential expression differences in the developing nervous system but makes other possible mechanisms more likely to explain the phenotypic variability in MCPH due to *ASPM* nonsense or frame-shift mutations. Potentially other variants in the genome (genetic modifiers) may alleviate or exacerbate the severity of the disease, resulting in the variability of phenotypic outcomes [26]. Especially in consanguineous families, differences in the segregation pattern of further rare homozygous variants could contribute to the phenotypic variability. In our reported family, this can be excluded, since only a very limited number of frequent and obviously nonpathogenic additional homozygous variants was observed (Table 2). Therefore other, potentially epigenetic mechanisms might contribute to this phenomenon.

## 5. Conclusions

The large phenotypic variability seen in patients with primary hereditary microcephaly due to *ASPM* mutations correlates well with the degree of structural brain abnormalities. The expression levels of the transcript for the truncated ASPM protein suggest that differences in the degree of structural cerebral changes cannot be explained by nonsense-mediated decay causing differences in *ASPM* expression.

## Figures and Tables

**Table 1:** Cognitive and disability assessment by rating scales.

Patient	II.2	II.3	II.4
RPM *	<55	<55	<55
KABC-II **	45	48	48
GASE ***	5	n.a.	n.a.
mRS ****	3	2	3
Barthel index	100	100	100
SINGER physical activities (%) *****	100	100	100
SINGER cognition (%) *****	45	55	48
SINGER household (%) *****	20	60	40
SINGER total (%) *****	74	80	76

* Raven’s Progressive Matrices (RPM) [8]; ** KABC-II–Kaufman Assessment Battery for Children Second Edition [9]; *** GASE scale—Global assessment of the severity of epilepsy [10]; **** modified Rankin scale (mRS) [12]; ***** SINGER scale [11]; n.a.—not applicable (no epilepsy).

**Table 2:** Homozygous and hemizygous variants with CADD * score > 20 detected in the index patient (II.2).

Gene Name	Genomic Position (GRCh 38)	Amino Acid Change	Allele Frequency ^#^	Homozygous/Hemizygous ^#^
*APP*	21:26000158G > A	p.Thr297Met	52/282,588	2
*TRIM67*	1:231206722G > A	p.Arg584Gln	144/277,698	1
*ASPM*	1:197103775_197103776del	p.Ile1826Serfs * 4	n.a.	n.a.
*LUZP4*	X:115289767C > G	p.Ser3Trp	160/204,902	2 / 92

* CADD—Combined Annotation-Dependent Depletion [19] ^#^ according to gnomAD database v2.1.1 [14]. n.a.—not applicable.
